# Supplementary material for: Sand-Related Factors Influencing Nest Burrowing Potential of the Sand Martins
Source: Animals (Basel). 2023 Nov 9;13(22):3463. doi: 10.3390/ani13223463 (PMC10668749; doi:10.3390/ani13223463)
Supplement: Supplementary file 1 [file animals-13-03463-s001.zip › animals-2652465-supplementary.pdf]

Table S1. Mixed Model Analyses of nest characteristics (dependent variables: tunnel depth, distance between tunnel holes, width of the entrance opening, and height of the entrance opening) and covariance parameter estimations between individual nest hole residuals and intercept variances (subject = [colony]; four colonies).

A) Dependent variable "Tunnel Depth"

Information Criteria

-2 Restricted Log Likelihood	612.342
Akaike's Information Criterion (AIC)	616.342
Hurvich and Tsai's Criterion (AICC)	616.500
Bozdogan's Criterion (CAIC)	623.081
Schwarz's Bayesian Criterion (BIC)	621.081

Fixed Effects

Type III Tests of Fixed Effects

Source	Numerator df	Denominator df	F	Sig.
Intercept	1	3	63.621	0.004

Estimates of Fixed Effects

Parameter	Estimate	Std. Error	df	t	Sig.	95% Confidence Interval	
						Lower Bound	Upper Bound
Intercept	53.612500	6.721502	3	7.976	0.004	32.221680	75.003320

Covariance Parameters

Estimates of Covariance Parameters

Parameter		Estimate	Std. Error	Wald Z	Sig.	95% Confidence Interval	
						Lower Bound	Upper Bound
Residual		112.863487	18.308875	6.164	<0.001	82.124046	155.108853
Intercept [subject = Colony]	Variance	175.071201	147.555509	1.186	0.235	33.557941	913.343448

B) Dependent variable "Distance to the between tunnel holes"

Information Criteria

-2 Restricted Log Likelihood	348.319
Akaike's Information Criterion (AIC)	352.319
Hurvich and Tsai's Criterion (AICC)	352.477
Bozdogan's Criterion (CAIC)	359.058
Schwarz's Bayesian Criterion (BIC)	357.058

Fixed Effects

Type III Tests of Fixed Effects

Source	Numerator df	Denominator df	F	Sig.
Intercept	1	3	55.82	0.005

Estimates of Fixed Effects

Parameter	Estimate	Std. Error	df	t	Sig.	95% Confidence Interval Lower Bound	95% Confidence Interval Upper Bound
Intercept	5.668750	0.758725	3	7.471	0.005	3.254148	8.083352

Covariance Parameters

Estimates of Covariance Parameters

Parameter		Estimate	Std. Error	Wald Z	Sig.	95% Confidence Interval Lower Bound	95% Confidence Interval Upper Bound
Residual		4.155428	0.674099	6.164	<0.001	3.023658	5.710825
Intercept [subject = Colony]	Variance	2.094885	1.880413	1,114	0.265	0.360665	12.167930

C) Dependent variable “Width of the entrance opening”

Information Criteria[a]

-2 Restricted Log Likelihood	203.0496
Akaike's Information Criterion (AIC)	207.496
Hurvich and Tsai's Criterion (AICC)	207.654
Bozdogan's Criterion (CAIC)	214.235
Schwarz's Bayesian Criterion (BIC)	212.235

Fixed Effects

Type III Tests of Fixed Effects

Source	Numerator df	Denominator df	F	Sig.
Intercept	1	3	701.014	<0.001

Estimates of Fixed Effects

						95% Confidence Interval	
Parameter	Estimate	Std. Error	df	t	Sig.	Lower Bound	Upper Bound
Intercept	6.000000	0.226615	3	26.477	<0.001	5.278811	6.721189

Covariance Parameters

Estimates of Covariance Parameters

						95% Confidence Interval	
Parameter		Estimate	Std. Error	Wald Z	Sig.	Lower Bound	Upper Bound
Residual		0.679934	0.110300	6,164	<0.001	0.494748	0.934437
Intercept [subject = Colony]	Variance	0.171420	0.167813	1.021	0.307	0.025164	1.167752

D) Dependent variable “Height of the entrance opening”

Information Criteria

-2 Restricted Log Likelihood	150.327
Akaike's Information Criterion (AIC)	154.327
Hurvich and Tsai's Criterion (AICC)	154.485
Bozdogan's Criterion (CAIC)	161.066
Schwarz's Bayesian Criterion (BIC)	159.066

Fixed Effects

Type III Tests of Fixed Effects

Source	Numerator df	Denominator df	F	Sig.
Intercept	1	3	2201.256	<0.001

Estimates of Fixed Effects

Parameter	Estimate	Std. Error	df	t	Sig.	95% Confidence Interval Lower Bound	95% Confidence Interval Upper Bound
Intercept	3.662500	0.078062	3	46.918	<0.001	3.414070	3.910930

Covariance Parameters

Estimates of Covariance Parameters

Parameter		Estimate	Std. Error	Wald Z	Sig.	95% Confidence Interval Lower Bound	95% Confidence Interval Upper Bound
Residual		0.367434	0.059606	6.164	<0.001	0.267360	0.504967
Intercept [subject = Colony]	Variance	0.006003	0.020124	0.298	0.765	8.414377E-6	4.283084

Table S2. Mixed Model Analyses of nest characteristics (dependent variables: tunnel depth, distance between tunnel holes, width of the entrance opening, and height of the entrance opening) and covariance parameter estimations between individual nest hole residuals and intercept variances (subject = lake site [LakeSiteNum]; two groups: lake-shore colonies [two colonies]; non-shore colonies [two colonies]).

A) Dependent variable "Tunnel depth"

Information Criteria

-2 Restricted Log Likelihood	663.267
Akaike's Information Criterion (AIC)	667.267
Hurvich and Tsai's Criterion (AICC)	667.425
Bozdogan's Criterion (CAIC)	674.006
Schwarz's Bayesian Criterion (BIC)	672.006

Fixed Effects

Type III Tests of Fixed Effects

Source	Numerator df	Denominator df	F	Sig.
Intercept	1	1.000	549.300	0.027

Estimates of Fixed Effects

						95% Confidence Interval	
Parameter	Estimate	Std. Error	df	t	Sig.	Lower Bound	Upper Bound
Intercept	53.612500	2.287500	1.000	23.437	0.027	24.547057	82.677943

Covariance Parameters

Estimates of Covariance Parameters

						95% Confidence Interval	
Parameter		Estimate	Std. Error	Wald Z	Sig.	Lower Bound	Upper Bound
Residual		243.613782	39.009425	6,245	<0.001	177,992016	333,428859
Intercept[subject=LakeSite]	Variance	4.374968	14.832283	0.295	0.768	0.005691	3363.335642

B) Dependent variable "Distance to the between tunnel holes"

Information Criteria

-2 Restricted Log Likelihood	349.172
Akaike's Information Criterion (AIC)	353.172
Hurvich and Tsai's Criterion (AICC)	353.330
Bozdogan's Criterion (CAIC)	359.911
Schwarz's Bayesian Criterion (BIC)	357.911

Fixed Effects

Type III Tests of Fixed Effects

Source	Numerator df	Denominator df	F	Sig.
Intercept	1	1.000	23,525	0.129

Estimates of Fixed Effects

Parameter	Estimate	Std. Error	df	t	Sig.	95% Confidence Interval Lower Bound	Upper Bound
Intercept	5.668750	1.168750	1.000	4.850	0.129	-9.181627	20.519127

Covariance Parameters

Estimates of Covariance Parameters

Parameter		Estimate	Std. Error	Wald Z	Sig.	95% Confidence Interval Lower Bound	Upper Bound
Residual		4.419151	0.707630	6.245	<0.001	3.228773	6.048395
Intercept[subject= LakeSiteNum]	Variance	2.621474	3.863606	0.679	0.497	0.145888	47.105473

C) Dependent variable "Width of the entrance opening"

Information Criteria

-2 Restricted Log Likelihood	202.463
Akaike's Information Criterion (AIC)	206.463
Hurvich and Tsai's Criterion (AICC)	206.620
Bozdogan's Criterion (CAIC)	213.201
Schwarz's Bayesian Criterion (BIC)	211.201

Fixed Effects

Type III Tests of Fixed Effects

Source	Numerator df	Denominator df	F	Sig.
Intercept	1	1	293.878	0.037

Estimates of Fixed Effects

						95% Confidence Interval	
Parameter	Estimate	Std. Error	df	t	Sig.	Lower Bound	Upper Bound
Intercept	6.000000	0.350000	1	17.143	0.037	1.552828	10.447172

Covariance Parameters

Estimates of Covariance Parameters

						95% Confidence Interval	
Parameter		Estimate	Std. Error	Wald Z	Sig.	Lower Bound	Upper Bound
Residual		0.694872	0.111269	6.245	<0.001	0.507696	0.951056
Intercept[subject= LakeSiteNum]	Variance	0.227628	0.346493	0.657	0.511	0.011522	4.496939

D) Dependent variable “Height of the entrance opening”

Information Criteria

-2 Restricted Log Likelihood	149.268
Akaike's Information Criterion (AIC)	153.268
Hurvich and Tsai's Criterion (AICC)	153.426
Bozdogan's Criterion (CAIC)	160.007
Schwarz's Bayesian Criterion (BIC)	158.007

Fixed Effects

Type III Tests of Fixed Effects

Source	Numerator df	Denominator df	F	Sig.
Intercept	1	1	858.490	0.022

Estimates of Fixed Effects

						95% Confidence Interval	
Parameter	Estimate	Std. Error	df	t	Sig.	Lower Bound	Upper Bound
Intercept	3.662500	0.125000	1	29.300	0.022	2.074224	5.250776

Covariance Parameters

Estimates of Covariance Parameters

						95% Confidence Interval	
Parameter		Estimate	Std. Error	Wald Z	Sig.	Lower Bound	Upper Bound
Residual		0.360737	0.057764	6.245	<0.001	0.263566	0.493733
Intercept[subject= LakeSiteNum]	Variance	0.022232	0.044218	0.503	,615	0.000451	1,096423

Table S3. Spearman's rho correlations. Significant correlations after Bonferroni corrections are indicated by bolding,

		Spearman's rho	p
Tunnel Depth (cm)	Distance to the between tunnel holes (cm)	-0.060	0.595
Tunnel Depth (cm)	Width of the entrance opening (cm)	-0.086	0.449
Tunnel Depth (cm)	Height of the entrance opening (cm)	-0.118	0.299
**Tunnel Depth (cm)**	**pH**	**0.484***	**< 0.001**
**Tunnel Depth (cm)**	**Electrical conductivity (EC)**	**0.321****	**0.004**
Tunnel Depth (cm)	Soil particle size (µm)	0.055	0.626
Distance between tunnel holes (cm)	Width of the entrance opening (cm)	-0.116	0.306
Distance between tunnel holes (cm)	Height of the entrance opening (cm)	-0.095	0.402
**Distance between tunnel holes (cm)**	**pH**	**-0.374*****	**<0 .001**
Distance between tunnel holes (cm)	Electrical conductivity (EC)	-0.114	0.314
Distance between tunnel holes (cm)	Soil particle size (µm)	-0.029	0.801
Width of the entrance opening (cm)	Height of the entrance opening (cm)	0.066	0.562
Width of the entrance opening (cm)	pH	0.189	0.093
**Width of the entrance opening (cm)**	**Electrical conductivity (EC)**	**0.353****	**0.001**
Width of the entrance opening (cm)	Soil particle size (µm)	0.090	0.427
Height of the entrance opening (cm)	pH	0.159	0.159
Height of the entrance opening (cm)	Electrical conductivity (EC)	0.048	0.673
Height of the entrance opening (cm)	Soil particle size (µm)	0.044	0.701
**pH**	**Electrical conductivity (EC)**	**0.458*****	**< 0.001**
pH	Soil particle size (µm)	-0.021	0.856
Electrical conductivity (EC)	Soil particle size (µm)	0.044	0.699

* p < 0.05, ** p < 0.01, *** p <0 .001

Table S4. Main data of the study.

Colony	Tunnel Depth (cm)	Distance to the between tunnel holes (cm)	Width of the entrance opening (cm)	Height of the entrance opening (cm)	pH	Electrical conductivity (EC)	soil particle size (µm)
1	55	4	5	3.5	8.72	140	106
1	49	5	5.5	4	8.34	141	106
1	33	7	7	4	8.32	136	106
1	32	9	7	4.5	8.49	140	425
1	43	11	7	3.5	8.66	141	425
1	46	5	5.5	4	8.71	143	106
1	70	8	5	4	8.54	139	106
1	51	9	8	3	8.34	141	425
1	45	8	6	4	8.72	137	425
1	59	9	6	3.5	8.52	142	106
1	34	6	6	3	8.72	140	20
1	37	7	5.5	3.5	8.36	141	106
1	48	5	6	3.5	8.41	145	425
1	43	11	4.5	2.5	8.54	144	106
1	68	6	5	3	8.62	136	106
1	43	11	7	4	8.34	148	106
1	70	7	6	3	8.49	135	106
1	45	7.5	5	3.5	8.54	141	425
1	34	9.5	5	2.5	8.29	142	106
1	33	7	6	3	8.47	140	20
2	65	3	6	2.5	8.76	127	106
2	62	5	6	3	8.71	122	106
2	65	5.5	5.5	5	8.8	124	106
2	70	2	5.5	4	8.39	121	106
2	45	3	6	3	8.72	120	425
2	70	10	6	3	8.76	127	106
2	56	6	5.5	3.5	8.6	123	425
2	49	5.5	5.5	3.5	8.74	120	425
2	35	7	5.5	4	8.72	119	425
2	61	5	4.5	2.5	8.73	120	20
2	47	9	6	4	8.75	119	425
2	50	8	6	3	8.9	118	425
2	47	9	6	4	8.8	120	106
2	51	2	5	2.5	8.77	120	425
2	61	10	5.5	4	8.75	122	106
2	63	4.5	5	3.5	8.76	116	20
2	52	7	4.5	4	8.7	117	425
2	63	9	4.5	4	8.8	120	106
2	42	6	5	5	8.75	120	106
2	61	5	4.5	4	8.77	121	106
3	66	6	6	4	9.14	294	106
3	74	7.5	5.5	3	9.35	287	425
3	75	2.5	6	5	9.16	291	106
3	67.5	4	6.5	4	9.42	294	106
3	79	5	6	3.5	9.29	287	106

3	70.5	6	7	3	9.14	284	106
3	69	5.5	7	3.5	9.16	282	425
3	72	3.5	6.5	3.5	9.25	290	20
3	69	3.5	6.5	4	9.17	288	425
3	63	6.5	7	3.5	9.17	284	106
3	79	5	6.5	4	9.3	284	425
3	81	4.5	7.5	3	9.22	288	425
3	67.5	5	6.5	4	9.3	291	106
3	74	6	5	3	9.32	286	106
3	69	8	6	4	9.32	279	20
3	103	3.5	6.5	3.5	9.38	279	106
3	68.5	3	8	4	9.33	285	106
3	59	2.5	6	3.5	9.14	284	425
3	67.5	5	5.5	5	9.12	288	425
3	55	5	5	4	9.28	290	106
4	42	5	6.5	4	8.9	141	425
4	39	2	7	3	8.77	138	425
4	26	1.5	7.5	5	8.8	140	425
4	34	5	6	4	8.9	142	425
4	43	8	7	3.5	8.6	138	106
4	54	3	6	3	8.8	139	106
4	26	2.5	7	3.5	8.6	140	20
4	40	5	8.5	3.5	8.8	142	425
4	38	3.5	6	4	8.7	137	106
4	61	6.5	5.5	3.5	8.74	143	106
4	44	4	6.5	4	8.78	137	425
4	40.5	6	5	3	8.76	139	425
4	33	4.5	4.5	4	8.78	139	20
4	66	2.5	6	4	8.8	141	106
4	25	3.5	6.5	4	8.9	138	425
4	35	5	7.5	3.5	8.76	140	425
4	37	2.5	7	4	8.79	142	425
4	43	3	5.5	4	8.77	137	106
4	38	7.5	7	5	8.72	138	106
4	43	2	5	4	8.78	137	106

Colony code 1:CV-1, **2:**CV-2, **3:** DMS, **4:** DMC
